# CHIP/STUB1 Ubiquitin Ligase Functions as a Negative Regulator of ErbB2 by Promoting Its Early Post-Biosynthesis Degradation

**DOI:** 10.3390/cancers13163936

**Published:** 2021-08-04

**Authors:** Haitao Luan, Tameka A. Bailey, Robert J. Clubb, Bhopal C. Mohapatra, Aaqib M. Bhat, Sukanya Chakraborty, Namista Islam, Insha Mushtaq, Matthew D. Storck, Srikumar M. Raja, Vimla Band, Hamid Band

**Affiliations:** 1 Eppley Institute for Research in Cancer and Allied Diseases, University of Nebraska Medical Center, Omaha, NE 68198, USA; h.luan@unmc.edu (H.L.); tabaile@uark.edu (T.A.B.); robert.clubb@novartis.com (R.J.C.); bmohapat@unmc.edu (B.C.M.); mstorck@unmc.edu (M.D.S.); srikumar.raja@northwestern.edu (S.M.R.); 2 Departments of Genetics, Cell Biology & Anatomy, College of Medicine, University of Nebraska Medical Center, Omaha, NE 68198, USA; aaqib.bhat@unmc.edu (A.M.B.); sukanya.chakraborty@unmc.edu (S.C.); namista.islam@unmc.edu (N.I.); 3 Department of Molecular Biology, College of Basic Medical Sciences, Jilin University, Changchun 130000, China; 4 Departments of Pathology & Microbiology, College of Medicine, University of Nebraska Medical Center, Omaha, NE 68198, USA; insha.mushtaq@unmc.edu; 5 Departments of Biochemistry & Molecular Biology, College of Medicine, University of Nebraska Medical Center, Omaha, NE 68198, USA; 6 Fred & Pamela Buffett Cancer Center, University of Nebraska Medical Center, Omaha, NE 68198, USA

**Keywords:** CHIP/STUB1, ubiquitin, ubiquitin ligase, E3, breast cancer, ErbB2, degradation, endoplasmic reticulum, Golgi, Trastuzumab, Bortezomib, pulse-chase

## Abstract

**Simple Summary:**

Overexpressed ErbB2/HER2 receptor drives up to a quarter of breast cancers. One aspect of ErbB2 biology that is poorly understood is how it reaches the cell surface following biosynthesis in the endoplasmic reticulum (ER). Here, the authors show that the CHIP (C-terminus of HSC70-Interacting protein)/STUB1 (STIP1-homologous U-Box containing protein 1) protein targets the newly synthesized ErbB2 for ubiquitin/proteasome-dependent degradation in the ER and Golgi, identifying a novel mechanism that negatively regulates cell surface expression of ErbB2. These findings provide one explanation for frequent loss of CHIP expression is ErbB2-overexpressing breast cancers. The authors further show that ErbB2-overexpressing breast cancer cells with low CHIP expression exhibit higher ER stress inducibility, and ER stress-inducing anticancer drug Bortezomib synergizes with ErbB2-targeted humanized antibody Trastuzumab to inhibit cancer cell proliferation. These new insights suggest that reduced CHIP expression may specify ErbB2-overexpressing breast cancers suitable for combined treatment with Trastuzumab and ER stress inducing agents.

**Abstract:**

Overexpression of the epidermal growth factor receptor (EGFR) family member ErbB2 (HER2) drives oncogenesis in up to 25% of invasive breast cancers. ErbB2 expression at the cell surface is required for oncogenesis but mechanisms that ensure the optimal cell surface display of overexpressed ErbB2 following its biosynthesis in the endoplasmic reticulum are poorly understood. ErbB2 is dependent on continuous association with HSP90 molecular chaperone for its stability and function as an oncogenic driver. Here, we use knockdown and overexpression studies to show that the HSP90/HSC70-interacting negative co-chaperone CHIP (C-terminus of HSC70-Interacting protein)/STUB1 (STIP1-homologous U-Box containing protein 1) targets the newly synthesized, HSP90/HSC70-associated, ErbB2 for ubiquitin/proteasome-dependent degradation in the endoplasmic reticulum and Golgi, thus identifying a novel mechanism that negatively regulates cell surface ErbB2 levels in breast cancer cells, consistent with frequent loss of CHIP expression previously reported in ErbB2-overexpressing breast cancers. ErbB2-overexpressing breast cancer cells with low CHIP expression exhibited higher endoplasmic reticulum stress inducibility. Accordingly, the endoplasmic reticulum stress-inducing anticancer drug Bortezomib combined with ErbB2-targeted humanized antibody Trastuzumab showed synergistic inhibition of ErbB2-overexpressing breast cancer cell proliferation. Our findings reveal new insights into mechanisms that control the surface expression of overexpressed ErbB2 and suggest that reduced CHIP expression may specify ErbB2-overexpressing breast cancers suitable for combined treatment with Trastuzumab and ER stress inducing agents.

## 1. Introduction

The ErbB family (ErbB1-4) of transmembrane receptor tyrosine kinases (RTKs) plays critical physiological roles [1,2,3]. ErbB1 (EGFR) and ErbB2 (HER2/Neu) drive oncogenesis in many human malignancies; ErbB2 overexpression, due to gene amplification and/or increased transcription, drives oncogenesis in up to a quarter of human breast cancer patients and specifies poor overall patient survival [4,5]. ErbB2 overexpression has been successfully exploited for therapeutic targeting with humanized monoclonal antibodies (e.g., Trastuzumab, Pertuzumab) and more recently with small molecule kinase inhibitors (e.g., Lapatinib), resulting in significant improvement of the treatment outcomes when added to conventional chemo-radiotherapy [6,7]. De novo as well as acquired resistance, however, has emerged as a major limitation to ErbB2-targetd therapy [8,9]. Newer insights into ErbB2 biology are required to open avenues to promote more effective and durable responses to targeted therapy of ErbB2-driven breast and other cancers.

ErbB2 is a transmembrane (TM) glycoprotein synthesized in the endoplasmic reticulum (ER), and as such is subject to ER quality control, which ensures that the newly synthesized proteins do not exit the ER until they are determined to be correctly folded and fully assembled [10,11,12]. Increase in the abundance of misfolded proteins triggers an unfolded protein response, one arm of which carries out the dislocation of misfolded/unassembled TM proteins into the cytosol for ubiquitin-dependent proteasomal degradation, a conserved process of ER-associated degradation (ERAD) [13]. Recent research showed that ErbB2+ breast cancers express higher levels of gene-encoding proteins in the ERAD pathway, potentially as a strategy to cope with ER stress caused by higher ER biosynthetic load from high abundance of ErbB2 and proteins in downstream pathways [14]. A subset of ER-synthesized proteins, which includes the ErbB family member ErbB3, also undergo an ER “quantity” control, which targets otherwise correctly folded proteins to ERAD, to maintain their physiological levels [15,16]. The folding of the luminal domains of TM proteins is mediated by ER luminal Hsp90-family chaperones, such as Hsp94, while the folding of the cytoplasmic domains is mediated by the Hsp90-Hsc70 chaperone (Hsc70 will be used here to collectively refer to both constitutive and inducible members of the Hsp70 family) [17]. Both pathways are hyperactive in ErbB2-overexpressing breast cancers and appear critical to maintain the oncogenic drive [13]. 

Hsp90-Hsc70 chaperone complex is a dual-purpose protein machine that promotes folding, but the Hsc70 component can also attain a pro-degradation conformation (regulated by co-chaperones) to facilitate the degradation of client proteins whose misfolding is sensed to be beyond restoration [4,18]. Studies of cystic fibrosis transmembrane-conductance regulator (CFTR) as a model TM protein illustrate this process and have established that ER quality control of the cytoplasmic domains of TM proteins requires the Hsp90-Hsc70 chaperone; in this process, the associated ubiquitin ligase (E3) CHIP/STUB1 functions to promote the pro-degradation state of Hsc70, leading to CFTR ERAD [19,20,21]. 

Newly synthesized RTKs, including EGFR and ErbB2, associate with Hsp90-Hsc70 in the ER and inhibition of Hsp90 with geldanamycin (GA) or its analogues (such as 17AAG) promotes rapid RTK degradation, indicating a requirement for Hsp90-Hsp70 complex to promote the folding of newly synthesized RTKs [22,23,24]. Distinct from other RTKs, however, ErbB2 remains Hsp90-associated even after its exit from the ER [25,26,27,28]. As we and others have shown, Hsp90 inhibition promotes the rapid ubiquitination and degradation of mature ErbB2 protein [24,29,30]. Notably, Hsp90 inhibitors exhibit selectively higher antitumor effects against ErbB2-overexpressing breast cancer cells and these effects are synergistic with ErbB2-targeted therapeutics Trastuzumab or Lapatinib [31]. Thus, increased Hsp90-Hsc70 chaperone function in ErbB2-overexpressing breast cancers is a co-driver of oncogenesis and a therapeutic target.

Since treatment of ErbB2+ breast cancer cells with Hsp90 inhibitors promotes rapid ubiquitination and proteasome-dependent degradation [23], identification of CHIP as a binding partner and inhibitor of the folding function of Hsc70 [32] suggested that it may serve as a mediator of Hsp90 inhibitor-induced ErbB2 degradation. Consistent with this idea, we and others observed that overexpression of CHIP enhanced the GA- or 17AAG-induced ubiquitination and degradation of ErbB2 [29,30]. However, ErbB2 degradation upon Hsp90 inhibition was unaffected in CHIP-null mouse embryonic fibroblasts (MEFs) [30]. Another study identified Cullin5 as an E3 ubiquitin ligase promoting ErbB2 ubiquitination and degradation in response to HSP90 inhibitors [33]. Thus, these studies clearly established that CHIP was not a mediator of acute degradation of mature ErbB2 (which is predominantly at the cell surface) upon Hsp90 inhibition [33]. However, despite a lack of involvement of CHIP in Hsp90 inhibitor-induced ErbB2 degradation, several recent lines of evidence support a critical involvement of CHIP in the regulation of ErbB2-driven oncogenesis. Reduced CHIP expression was seen in breast cancer patient tumors [34,35,36] and in ErbB2-overexpressing and Triple Negative Breast Cancer (TNBC) cell lines [35]. Ectopic CHIP expression in ErbB2-overexpressing breast cancer cell lines suppressed the in vitro oncogenic traits and in vivo xenograft tumor growth [35]. Thus, CHIP exerts strong negative constraints on ErbB2-driven oncogenesis. One pro-oncogenic mechanism unleashed by the loss of CHIP involves CHIP interaction with and ubiquitination and degradation of a transcription factor Myeloid Zinc Finger 1 (MZF1), reduced expression of cathepsins B and L and reduced extracellular matrix degradation, migration, and invasion [35]. In non-ErbB2-overexpressing breast cancer cell lines, loss of CHIP expression was shown to increase the expression of transcriptional co-regulator SRC-3, with increased expression of pro-oncogenic proteins such as Smad2 and twist, to promote metastasis [34]. However, how loss of CHIP in ErbB2-overexpressing breast cancers might regulate ErbB2 itself is unclear. In this study, we demonstrate that newly synthesized ErbB2 is a direct target of CHIP-mediated ubiquitination and degradation through a post-biosynthetic degradation pathway in the ER and potentially Golgi apparatus, with loss of CHIP expression seen in ErbB2-overexpressing breast cancer cell lines promoting ErbB2 surface expression.

## 2. Materials and Methods

### 2.1. Cell Lines and Medium

ErbB2-overexpressing breast cancer cell lines SKBR3 and BT474 (ATCC, Manassas, Virginia, USA) were cultured in complete α-MEM medium with 10% fetal bovine serum, 10 mM HEPES, 1 mM each of sodium pyruvate, nonessential amino acids, and glutamine, 50 µM 2-ME, and 1% penicillin/streptomycin (Life Technologies, Carlsbad, CA, USA). ErbB2-overexpressing breast cancer cell line 21MT1 cells generated in our laboratories [37], were cultured in α-HE medium (α-MEM medium supplemented with 1 μg/mL hydrocortisone and 12.5 ng/mL epidermal growth factor (Sigma-Aldrich, St. Louis, MO, USA). Cell lines were determined to be mycoplasma-free using a commercial PCR-based kit (cat. G238, Applied Biological Materials Inc., Richmond, BC, Canada).

### 2.2. Antibodies and Reagents

The following antibodies were used for immunoblotting: Ubiquitin—monoclonal antibody P4D1 from Cell signaling, Denver, PA, USA; ErbB2—monoclonal antibody (cat. 554299) from BD-Pharmingen, San Jose, CA, USA; Phospho-Akt (S473) rabbit antibody (cat. 9271S), total Akt rabbit antibody (cat. 4691S), GRP78 rabbit antibody (cat. 3177S), Phospho-eIF2α (Ser51) rabbit mAb (cat. 3398S), PERK rabbit monoclonal antibody (cat. 5683s) from Cell signaling, Danvers, MA; Hsc70 (B-6) antibody from Santa Cruz Biotechnology, Santa Cruz, CA, USA; CHIP—rabbit anti-serum made in the laboratory through Covance Research Products, Denver, PA, USA. ErbB2 immuno-staining was carried out using the AF1129 antibody from R&D Systems, Minneapolis, MN, USA, or Alexa Fluor-488 or 647-conjugated mouse anti-human ErbB2 (CD340) monoclonal antibody (clone 24D2; cat. 324,410 and 3,244,412, respectively) from BioLegend Inc., San Diego, CA, USA, with Alexa Fluor -488 or 647 mouse mAb IgG1 (MOPC-21) (cat. 400,129 and 400,130 from BioLegend) as controls. Secondary antibodies for immunostaining included Alexa Fluor 488 donkey anti-goat IgG for ErbB2 from Invitrogen Molecular Probes, Eugene, OR, USA. ER marker Calnexin and Golgi marker GM130 staining was carried out using antibodies from Cell signaling, Danvers, MA, USA (cat. 2679S for calnexin; cat. 12480S for GM130) with Alexa Fluor 594 rabbit antibody (cat. A-11037, Thermo Fisher, Waltham, MA, USA). Trastuzumab (Herceptin, obtained from UNMC Hospital Pharmacy) was dissolved in Phosphate Buffered Saline (PBS). Brefeldin A (cat. B7651, Sigma-Aldrich, St. Louis, MO, USA), thapsigargin (cat. T9033, Sigma-Aldrich, St. Louis, MO, USA) and Bortezomib (cat. 7282, Tocris Bioscience, Minneapolis, MN, USA) were dissolved in DMSO. Lambda protein phosphatase (cat. P753S) was from New England BioLabs, Ipswich, MA, USA.

### 2.3. Protein Lysis and Quantification

Cells were either lysed in Radio-Immuno-Precipitation Assay (RIPA) lysis buffer (50 mM Tris pH 7.5, 150 mM NaCl, 1% Triton-X-100, 0.05% deoxycholate, 0.1% Sodium Dodecyl Sulfate (SDS)) with or in Triton-X-100 lysis buffer (50 mM Tris pH 7.5, 150 mM NaCl, 0.5% Triton-X-100), 1 mM PMSF, 10 mM NaF, and 1 mM sodium orthovanadate. Lysates were rocked at 4 °C for a minimum of 1 h, centrifuged at 13,000 rpm for 20 min at 4 °C in a bench-top micro-centrifuge and supernatants were transferred to fresh tubes. Protein concentrations of samples were estimated using the Bicinchoninic acid (BCA) assay kit (Thermo Fisher Scientific, Rockford, IL, USA) or the Bradford assay reagent (Bio-Rad Laboratories, Hercules, CA, USA) using bovine serum albumin (BSA) as a standard.

### 2.4. FACS Analysis

SKBR3 and 21MT1 cells were seeded at 10^5^ cells per well of six-well plates (triplicates per condition) and grown in regular medium until 50–80% confluent. Cells were rinsed with ice-cold PBS and released from dishes with trypsin-EDTA (Life Technologies, Carlsbad, CA, USA). Trypsinization was stopped by adding soybean trypsin inhibitor (Life Technologies, Carlsbad, CA, USA). Cell suspensions were transferred to Eppendorf tubes and washed three times in ice-cold FACS buffer (1% bovine serum albumin in PBS). Live cells were stained with Alexa Fluor-488 conjugated mouse anti-human ErbB2 (CD340) monoclonal antibody (clone 24D2; cat. 324410) from BioLegend Inc., San Diego, CA, USA, with Alexa Fluor-488 mouse mAb IgG1 (MOPC-21) (cat. 400,129 from BioLegend) as control. FACS analyses were performed on a Becton Dickinson LSRII instrument, and data were analyzed using FlowJo software (https://www.flowjo.com/learn/flowjoportal, accessed on 16 July 2021).

### 2.5. Immunoprecipitation (IP) Reactions

Cells were lysed in RIPA buffer. Following the total protein estimation of the lysate samples, the optimized ratio of total lysate and antibody was incubated on a rocker overnight at 4 °C. An amount of 200 μL of 10% Protein A Sepharose (PAS, GE Healthcare, Chalfont St. Giles, UK) beads (washed with RIPA buffer) were added to each IP sample and rocked at 4 °C overnight. Following this incubation, the samples were centrifuged for 5 min at 13,000 rpm at 4 °C, the supernatant was removed to a separate tube, and the beads were washed five times with 1 mL of RIPA buffer. Then, 100 μL of 2X sample buffer (10% glycerol, 3% SDS, 0.02% bromophenol blue, 0.125 M Tris pH 6.8) was added to each sample, heated at 95 °C for 3–5 min, resolved by SDS-Polyacrylamide (Bio-Rad) Gel Electrophoresis (PAGE), transferred to Polyvinylidene fluoride (PVDF) membrane (Bio-Rad Laboratories, Hercules, CA, USA), and subjected to Western blotting.

### 2.6. Confocal Immunofluorescence Microscopy

Cells were grown on glass coverslips inside the wells of a 24-well tissue culture plate. After the completion of an experiment, cells were fixed in 4% paraformaldehyde (PFA) in PBS for 20 min. The PFA solution was removed, and the cells were permeabilized for 20 min in immunofluorescence (IF) buffer (10% FBS, 0.2% BSA, and 0.05% saponin in PBS). The cells were then stained with primary antibodies overnight at 4 °C followed by three 10 min washes in PBS. The cells were then incubated with the appropriate secondary antibodies for 1 h (diluted 1:500 in the IF buffer), followed by three 10 min washes in PBS. In preparation for confocal microscopy, PBS was removed, and the coverslips were mounted onto glass microscope slides with Vectashield mounting medium (Vector Laboratories, Burlingame, CA, USA), containing 4′, 6-diamidino-2-phenylindole (DAPI) to stain the nuclei. The images were captured using a Zeiss LSM710 confocal microscope. The Pearson coefficient of colocalization was determined using FIJI (package of ImageJ, NIH, Bethesda, MD, USA; https://fiji.sc/ accessed on 16 July 2021).

### 2.7. Transfection and Plasmids

The XtremeGENE 9 transfection reagent was from Roche Applied Science (Indianapolis, IN, USA); Fluorescent Golgi marker plasmid pmTurquoise2-Golgi was a gift from Dorus Gadella (Addgene plasmid # 36205; https://www.addgene.org/36205/; accessed on 2012 Mar 20; RRID: Addgene_36205).

### 2.8. ^35^S-Methionine/Cysteine Pulse Labeling Followed by Chase (Pulse-Chase)

Cells were grown in 10 cm dishes until 60–70% confluent. The medium was removed, cells were washed three times with PBS, and incubated with methionine- and cysteine-free DMEM medium (cat. 21013-024, Life technologies, Carlsbad, CA, USA) for 30 min at 37 °C. A ^35^S-labeled methionine/cysteine mixture (cat. NEG772, Perkin Elmer, Waltham, MA, USA) was added to a final concentration of 0.2 mCi/mL. After 20 min, cells were washed three times with cold PBS and chased with complete DMEM medium supplemented with 100-fold excess unlabeled methionine and cysteine for various time points with or without brefeldin-A treatment. 

### 2.9. Anchorage-Independent Growth on Soft Agar

2500 cells were seeded in 0.35% soft agar on top of 0.6% soft agar layer in complete medium in 6-well plates. After two weeks, cells were stained with crystal violet and imaged under a phase contrast microscope. The colonies were enumerated using the Image J software (NIH, Bethesda, MD, USA; https://imagej.nih.gov/ij/, accessed on 16 July 2021).

### 2.10. Statistical Analysis

Statistical analysis was performed using the SPSS 16.0 statistical software (SPSS Inc., Chicago, IL, USA). Group comparison analysis was performed using Student’s *t* test. *p* value (two-sided) of <0.05 was considered significant.

## 3. Results

### 3.1. CHIP Regulates Cell Surface ErbB2 Levels

**Figure 1:**
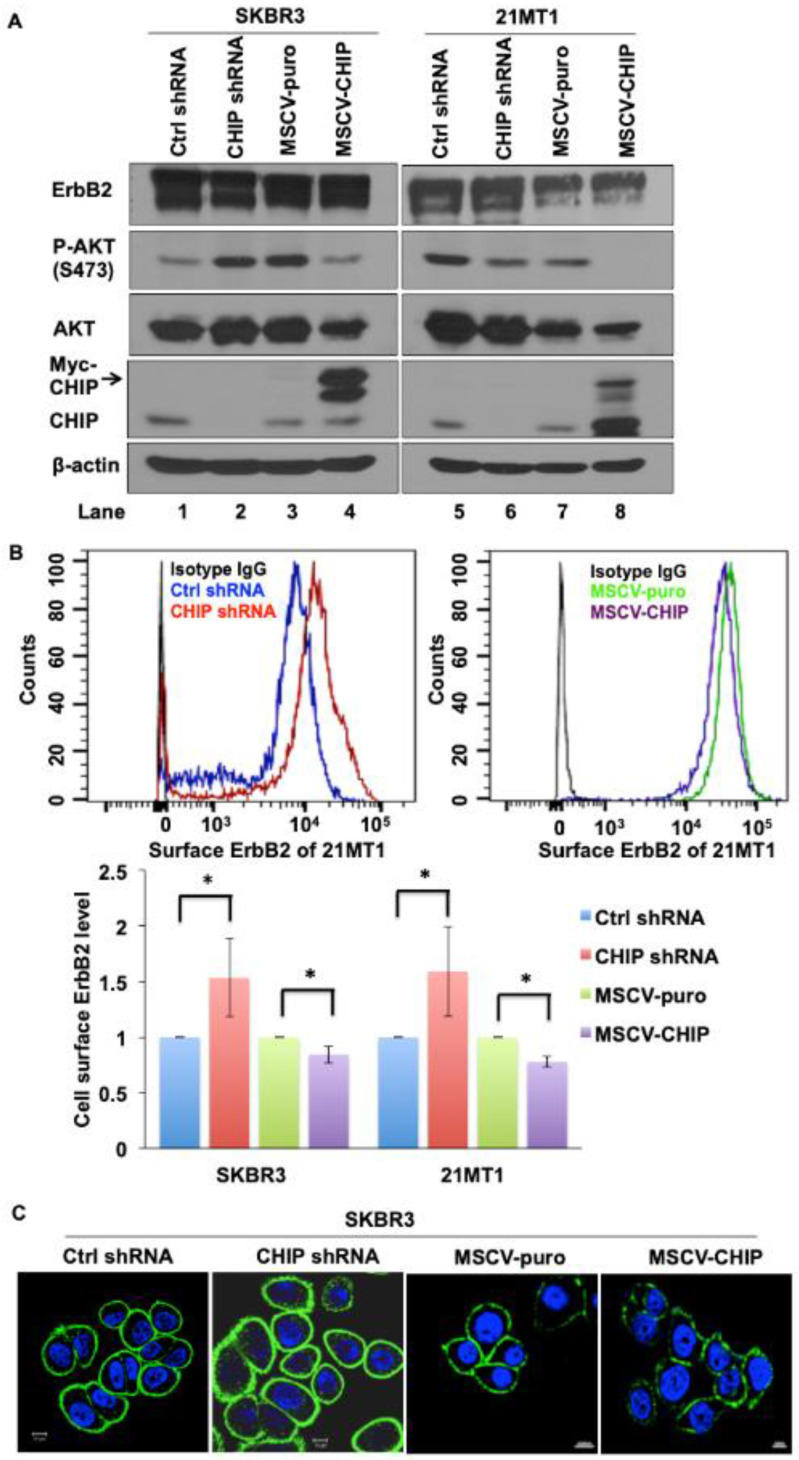
CHIP regulates cell surface ErbB2 levels. (**A**) Western blot analysis of SKBR3 and 21MT1 cell lysate for CHIP, ErbB2, phospho-Akt and total Akt levels; β-actin was used as a loading control. (**B**) SKBR3 and 21MT1 cell surface ErbB2 level was determined by FACS analysis. Representative FACS profiles are shown in top panel. Quantification of surface ErbB2 levels (based on relative change in mean fluorescence intensity with control shRNA as 1) is shown in the bottom panel. Data represent mean ± SEM, *n* = 3. * *p* < 0.05. (**C**) SKBR3 control shRNA, CHIP shRNA, MSCV-puro and MSCV-CHIP cells were seeded on glass coverslips, fixed, and stained with anti-ErbB2 antibody followed by staining with the corresponding fluorescent secondary conjugates. Coverslips were mounted with anti-fade containing DAPI and images were acquired under a Zeiss LSM 710 confocal microscope.

To examine the molecular mechanisms underlying CHIP-mediated ErbB2 degradation, we developed stable control and CHIP knockdown (KD) versions of ErbB2-overexpressing breast cancer cell lines SKBR3 and 21MT1 and used these along with their previously established vector control and Myc-tagged CHIP overexpressing transfectants [37]. We confirmed the reduction in CHIP levels in KD lines and the expression of a slower-migrating Myc-tagged CHIP in overexpressing lines (Figure 1A and Figure S1). The overall levels of ErbB2 detected by Western blotting did not show a consistent change upon stable CHIP KD or overexpression nor were consistent differences in downstream signaling molecule phospho-AKT seen upon CHIP KD under regular conditions of growth (Figure 1A and Figure S1). However, FACS analyses showed that cell surface ErbB2 levels increased in both SKBR3 and 21MT1 CHIP KD cells compared with control cells. Reciprocally, surface ErbB2 levels were decreased in SKBR3 and 21MT1 cells with CHIP overexpression when compared with puromycin-resistant vector control cells (Figure 1B). Additionally, higher surface ErbB2 staining was seen in CHIP KD cells compared to control cells when analyzed by immunofluorescence imaging (Figure 1C). These results suggest that CHIP negatively regulates the cell surface levels of ErbB2 protein in ErbB2-overexpressing breast cancer cell lines. 

### 3.2. CHIP Promotes the Ubiquitination and Degradation of ErbB2 during Its Post-Biosynthesis Maturation

As CHIP is an E3 ubiquitin ligase, we asked whether ErbB2 was a direct target of CHIP in the absence of any stimulus, such as Hsp90 inhibition [24,29,30], that induces ErbB2 degradation. Previous reports demonstrated that CHIP could regulate protein quality control in the ER, with CFTR as a well-studied example, as well as in the cytoplasm [19]. We therefore asked whether CHIP plays a role in ErbB2 quality/quantity control since ErbB2 overexpression could create opportunities for increased mis-folding of the newly synthesized protein.

**Figure 2:**
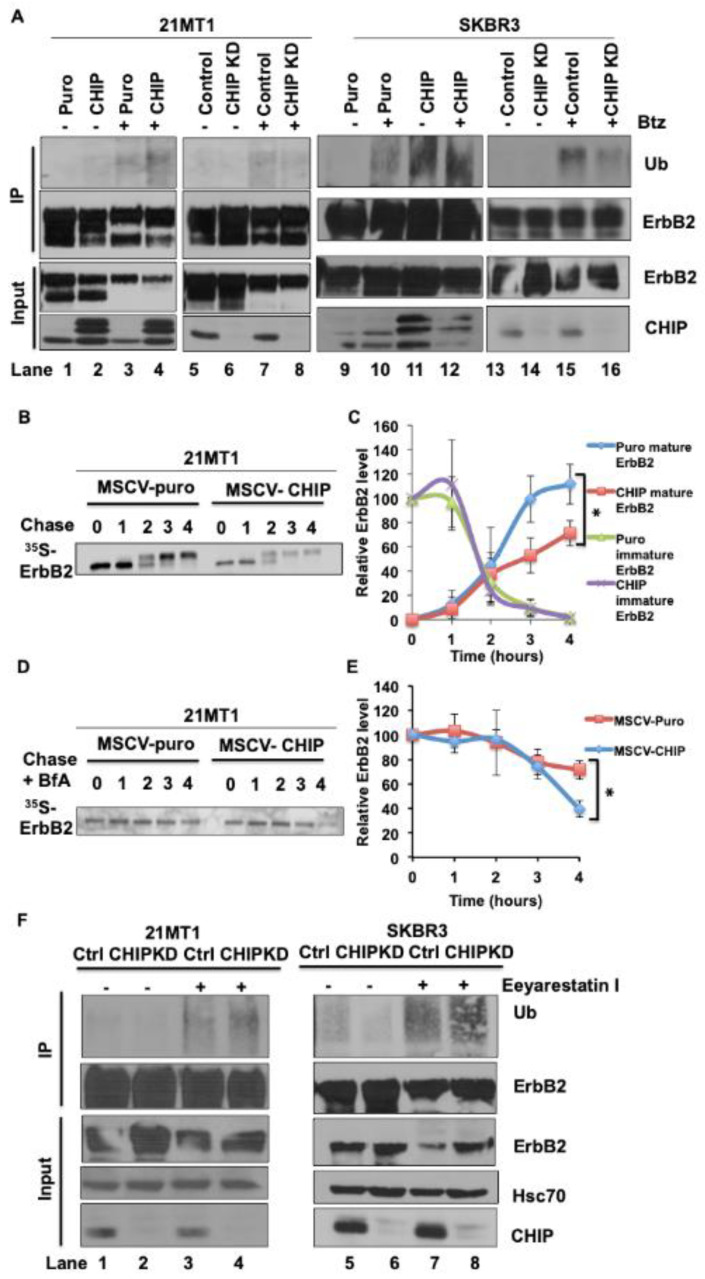
CHIP elevates the basal ubiquitination of ErbB2 during its maturation. (**A**) 21MT1 and SKBR3 cells were seeded in 10 cm dishes and incubated with or without proteasome inhibitor Bortezomib (1 μM) for 4 h. Cleared lysates (1 mg protein) of the indicated cells were subjected to ErbB2 immunoprecipitation using Trastuzumab (5 μg/mL). Ubiquitinated ErbB2 signals were detected by anti-ubiquitin immunoblotting (upper panel). ErbB2 and CHIP levels in whole cell lysates are shown in the lower panel. (**B**) 21MT1 cells were pulse-labeled for 20 min with [^35^S]-methionine/cysteine and were then chased with excess (100-fold) unlabeled methionine/cysteine-containing medium. Cleared lysates from cells harvested at the indicated time points were used for immunoprecipitation with anti-ErbB2 antibody (Trastuzumab), and immunoprecipitates were resolved by SDS-PAGE and visualized by autoradiography. (**C**) Relative ErbB2 level quantification in B. Data represent mean ± SEM, *n* = 3, * *p* < 0.05. (**D**) 21MT1 cells were pulse-labeled for 20 min with [^35^S] methionine-cysteine and chased with excess unlabeled methionine-cysteine medium in the presence of Brefeldin A (1 μg/mL). Cleared lysates from cells harvested at the indicated time points were immunoprecipitated with anti-ErbB2 antibodies, followed by autoradiography. (**E**) Relative ErbB2 level quantification in (**C**). Data represent mean ± SEM, *n* = 3, * *p* < 0.05. (**F**) 21MT1 or SKBR3 cells were seeded in 10-cm dishes and incubated with or without Eeyarestatin I (1 μM) for 4 h. Cleared lysates (1 mg protein) of the indicated cells were subjected to ErbB2 immunoprecipitation using Trastuzumab (5 μg in 1 mL). Ubiquitinated ErbB2 signals were detected by anti-ubiquitin immunoblotting (upper panel). ErbB2 and CHIP levels in whole cell lysates are shown in the lower panel.

To address the potential of CHIP to serve as an E3 ubiquitin ligase towards ErbB2 in ErbB2-overexpressing breast cancer cells, we performed immunoprecipitation analyses in both CHIP-overexpressing vs. empty vector control and CHIP shRNA knockdown vs. control shRNA-expressing ErbB2-overexpressing breast cancer cell lines. CHIP overexpression increased the basal ErbB2 ubiquitination even without treatment with the proteasome inhibitor Bortezomib while ubiquitin signals in control cells were barely detectable (lane 2 vs. 1, lane 9 vs. 11, Figure 2A and Figure S2A). Proteasome inhibition elevated the ErbB2 ubiquitination signals with substantially higher signals in CHIP-overexpressing cells when compared to control cells (lane 3 vs. 4, lane 10 vs. 12, Figure 2A and Figure S2A). CHIP KD decreased the level of ErbB2 ubiquitination upon proteasome inhibitor Bortezomib treatment compared to control shRNA cells (lane 7 vs. 8, lane 15 vs. 16, Figure 2A and Figure S2A). These data demonstrated that CHIP itself could function as an E3 ubiquitin ligase to target ErbB2 for proteasomal degradation in the absence of Hsp90 inhibition. However, the total ErbB2 level was not consistently changed when CHIP was KD or overexpressed in ErbB2-overexpressing cells (Figure 1A and Figure S1). One possible explanation for this discrepancy was that ectopic-CHIP expression was not high enough relative to ErbB2 in these ErbB2-overexpressing cell lines to clear all the ErbB2 protein; alternatively, CHIP may only target a pool of ErbB2 bound for delivery to the cell surface, hence accounting for the results of FACS analyses which showed a significant impact of manipulating CHIP levels on surface ErbB2 expression (Figure 1B). Given the role of CHIP in regulating the ubiquitination and degradation of newly synthesized CFTR [19,21,38], we reasoned that CHIP-dependent ubiquitination of ErbB2 may reflect targeting of the newly synthesized pool of ErbB2. To address this possibility, we performed ^35^S-methionine/cysteine pulse-chase experiments to investigate whether CHIP overexpression affected the stability and maturation of newly synthesized ErbB2. As expected [30], the major radiolabeled immature precursor species of ErbB2 observed after pulse-labeling (0 chase time) in control cells was converted to a mature form with higher molecular weight after chase (Figure 2B and Figure S2B); this species reflects the post-ER glycosylated form of ErbB2 that is eventually transported to the cell surface [30]. CHIP-overexpressing 21MT1 cells had a similar maturation pattern as the control cells; however, the intensity of the mature ErbB2 form in CHIP-overexpressing 21MT1 cells was reduced relative to the intensity of the initial precursor form compared to the pattern in control 21MT1 cells (Figure 2B,C and Figure S2B). These results are consistent with a proportion of the newly synthesized ErbB2 being targeted for degradation in a CHIP-dependent manner. As CHIP-dependent degradation of newly synthesized CFTR occurs in the ER, through ERAD, we asked whether a similar process accounts for CHIP-dependent degradation of newly synthesized ErbB2 in ErbB2-overexpressing breast cancer cells. To address this, we performed ^35^S-methione/cysteine pulse-chase experiments on 21MT1 puro vs. 21MT1-CHIP cell lines pretreated with brefeldin A (BfA), an inhibitor of the transport of newly synthesized membrane proteins from ER to Golgi apparatus [39]. Under BfA treatment, newly synthesized ErbB2 would be expected to remain in an immature form in the ER instead of maturing in the Golgi for transport to the cell surface. As expected, BfA treatment for 4 h efficiently blocked the appearance of the mature form of newly synthesized ErbB2 (Figure 2D,E and Figure S2C), as seen by the lack of a high-molecular-weight species that is detected in the absence of BfA (Figure 2B). Notably, CHIP-overexpressing 21MT1 cells showed a more pronounced loss of radiolabeled ErbB2 compared to that in puro control 21MT1 cells (Figure 2D and Figure S2C). This result supports the conclusion that CHIP targets the newly synthesized, immature form of ErbB2 for degradation in the ER. To assess whether the CHIP-dependent ErbB2 degradation involves the ERAD pathway, we used Eeyarestatin I [40], a potent inhibitor of ERAD through its ability to inhibit the AAA ATPase Valosin-Containing Protein (VCP)/p97, known to regulate the alternate fate of client proteins through its interactions with ubiquitin ligases and deubiquitinases [41]. As shown in Figure 2E (Figure S2D), Eeyarestatin I treatment elevated ErbB2 ubiquitination in both control and CHIP knockdown cells, which was similar to the effect of treatment with the proteasome inhibitor Bortezomib (Figure 2A). Unexpectedly, however, ErbB2 ubiquitination levels in CHIP knockdown cells upon Eeyarestatin I treatment were higher than in similarly treated control shRNA-expressing cells, and Eeyarestatin I treatment led to reduction in ErbB2 levels in all cases. These results suggest that VCP/p97 targets ErbB2 to additional E3 ubiquitin ligases and associated deubiquitinases to regulate its fate. One potential E3 candidate for such a role is Cullin 5, previously identified to promote ErbB2 ubiquitination and degradation in response to HSP90 inhibitors [33]. While our results support a role for the ERAD pathway is regulating the fate of newly synthesized ERB2, these results do not clarify whether the VCP/p97-depndent ERAD in fact mediates the CHIP-dependent post-synthesis turnover of ErbB2 at the level of ER we identified above (Figure 2B and Figure S2B). 

### 3.3. CHIP Overexpression Promotes the Intracellular Retention of ErbB2 in the Golgi 

**Figure 3:**
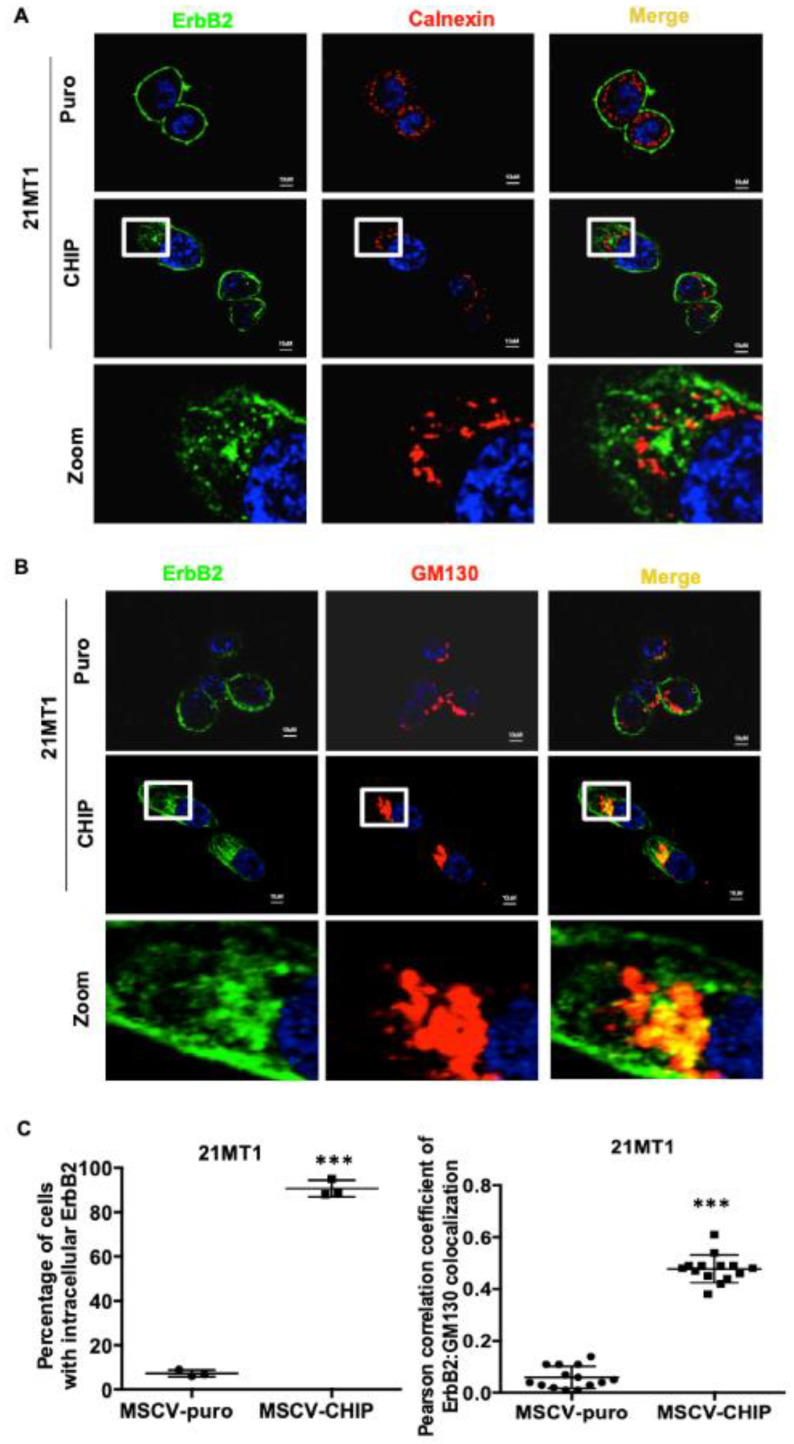

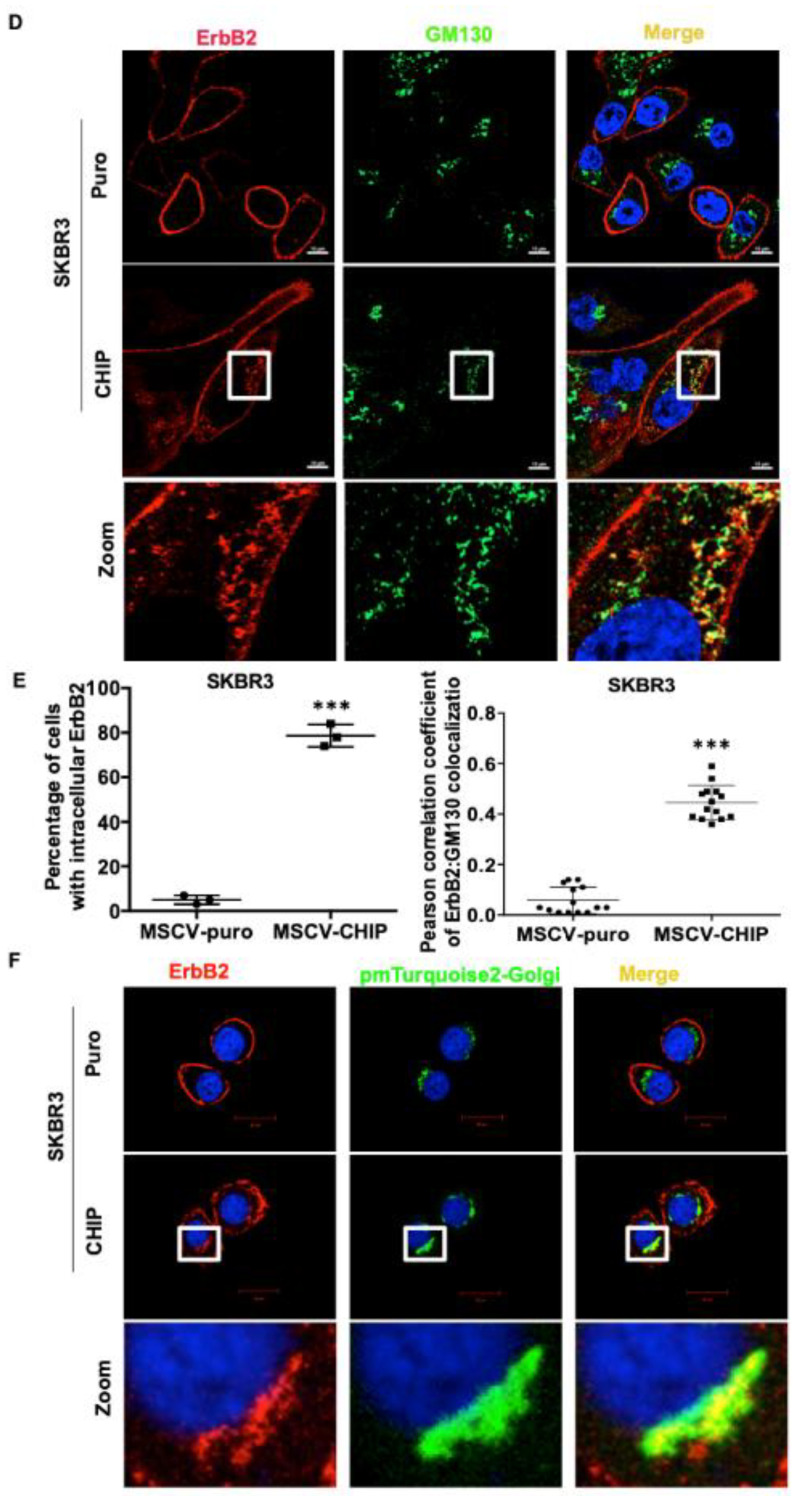
CHIP overexpression leads to increased ErbB2 retention in the Golgi. (**A**) 21MT1-puro and 21MT1-CHIP cell lines were seeded on glass coverslips for 48 h. Cells were fixed and stained with anti-ErbB2 (green) and anti-calnexin (ER marker) (red) antibodies followed by staining with the corresponding fluorescent secondary conjugates. Coverslips were mounted with anti-fade containing DAPI and images were acquired under a Zeiss LSM 710 confocal microscope. (**B**) 21MT1-puro and 21MT1-CHIP cell lines were seeded on coverslips, fixed, and stained with anti-ErbB2 (green) and anti-GM130 (red) antibody followed by incubation with secondary fluorescent conjugate as in A. Zoom images of selected representative area are shown in (**A**,**B**). (**C**) Quantification of percentage of cells with intracellular ErbB2 staining in A and B (left panel) and Pearson’s Coefficient of Colocalization between ErbB2 and GM130 in (**B**). Data represent mean ± SD, *n* = 3, *** *p* < 0.001. Pearson correlation coefficient was analyzed using FIJI function of ImageJ. (**D**) SKBR3-puro and SKBR3-CHIP cell lines were seeded on coverslips, fixed, and stained with anti-ErbB2 (red) and anti-GM130 (green) antibodies, followed by the respective fluorescent secondary conjugates. Coverslips were mounted with anti-fade containing DAPI and images were acquired under a Zeiss LSM 710 confocal microscope. Zoom images of selected representative area are shown. (**E**) Quantification of percentage of cells with intracellular ErbB2 staining (left panel) and Pearson’s Coefficient of Colocalization between ErbB2 and GM130 presented in (**D**). Data represent mean ± SD, *n* = 3, *** *p* < 0.001. Pearson correlation coefficient was analyzed using FIJI function of ImageJ. (**F**) SKBR3-puro and SKBR3-CHIP cell lines were seeded on coverslips and transiently transfected with pmTurquoise2-Golgi vector (shown in green) as a marker of the Golgi apparatus. Cells were fixed and stained with anti-ErbB2 antibody followed by the fluorescent secondary conjugate (red). Coverslips were mounted with anti-fade containing DAPI and images were acquired under a Zeiss LSM 710 confocal microscope. Zoom images of selected representative area are shown.

Since CHIP overexpression promoted the destabilization of immature ErbB2, this suggests that CHIP may function at the ER to prevent the incorrectly folded, newly synthesized ErbB2 from being exported to the cell surface and facilitating its early post-synthesis degradation. To explore this notion further, we carried out ErbB2 immunostaining in stably CHIP-overexpressing vs. control 21MT1 cells. In stably CHIP-overexpressing 21MT1 cells, we observed increased intracellular ErbB2 while little intracellular ErbB2 was seen in control cells (Figure 3A,C). Under these conditions, the intracellular ErbB2, however, did not co-localize with the ER marker calnexin (Figure 3A). Recent research has revealed an endosome and Golgi-associated degradation (EGAD) pathway in Saccharomyces cerevisiae for selective membrane protein degradation [42]. Based on this, we examined whether intracellular-localized ErbB2 in stably CHIP-overexpressing 21MT1 cells may reside in the Golgi. Indeed, the intracellular ErbB2 pool in these cells co-localized with the Golgi marker GM130 [43] (Figure 3B,C). To further validate the Golgi localization of intracellular ErbB2, we performed the colocalization analyses in SKBR3 cells either staining for endogenous Golgi marker GM130 or by transiently expressing a plasmid coding for a Golgi-localized pmTurquoise2-Golgi fluorescent probe [44]. We observed more intracellular ErbB2 staining in CHIP overexpressing SKBR3 cells and intracellular ErbB2 co-localized with Golgi marker GM130 (Figure 3D,E). The ectopic pmTurquoise2-Golgi marker also co-localized with intracellular ErbB2 in CHIP-overexpressing SKBR3 cells while no co-localization was observed in control cells (Figure 3F).

### 3.4. Low Expression of CHIP Sensitizes ER Stress Inducer Mediated Tumor Cell Growth Inhibition

**Figure 4:**
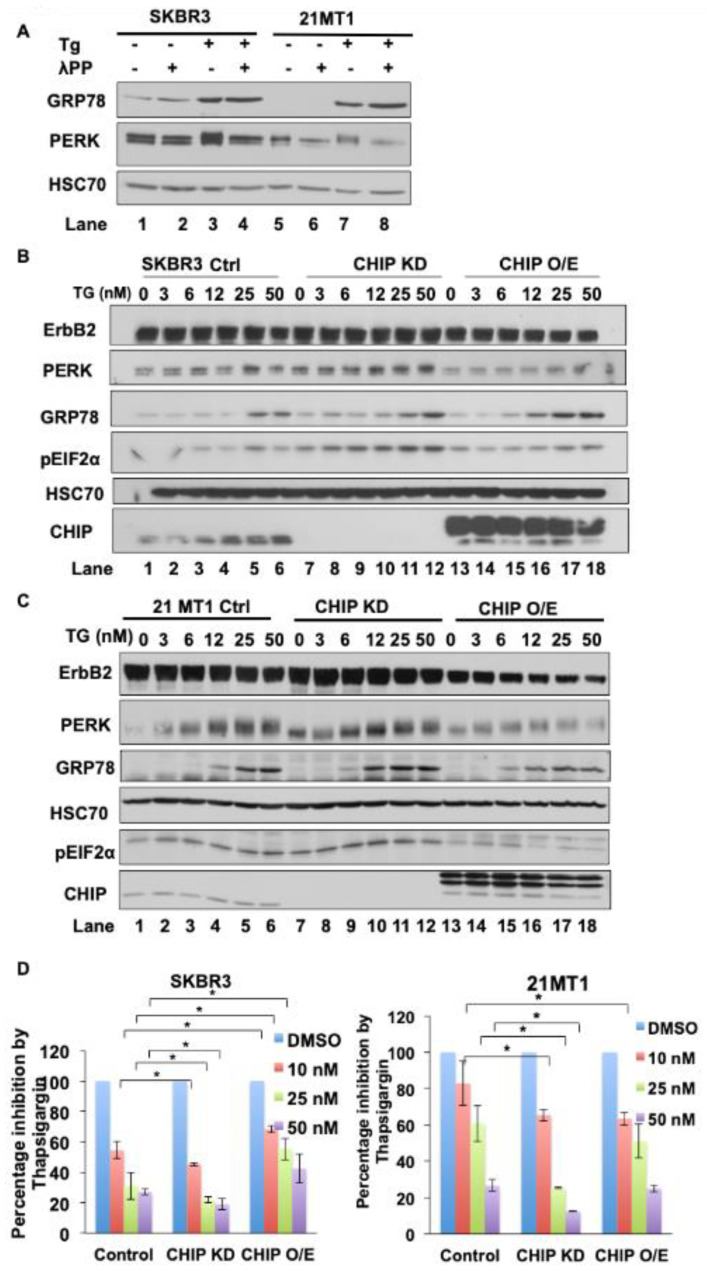
Reduced expression of CHIP sensitizes ErbB2-overexpressing breast cancer cell lines to ER stress. (**A**) SKBR3 and 21MT1 cells were seeded in six-well plates and incubated with ER stress inducer thapsigargin (50nM) for 24 h. Cleared lysates (50 μg protein/lane) were left untreated or treated with lambda protein phosphatase (400U) for 30 min and then subjected to Western blotting for GRP78, PERK and Hsc70 (loading control). (**B**,**C**) SKBR3 (**B**) and 21MT1 (**C**) cells were seeded in six-well plates and incubated with indicated concentrations of thapsigargin for 24 h. Cleared lysates (50 μg/lane) were subjected to Western blotting for ErbB2, GRP78, phospho-eIF2α, PERK, CHIP and Hsc70 (loading control). (**D**) SKBR3 and 21MT1 cells were seeded in soft agar (0.6% bottom layer, 0.35% top layer) in six-well plates and treated with the indicated concentrations of thapsigargin for 2 weeks. Data represent mean ± SD, *n* = 3, * *p* < 0.05.

The cellular demands for protein quality control are elevated in cancer cells as their higher metabolic needs create elevated ROS levels and the increased protein synthetic load requires additional protein unfolding capacity [10]. Since CHIP is known to serve as a key regulator of protein quality control [32,45], it is reasonable to posit that lower levels of CHIP in ErbB2-overexpressing breast cancer cells could promote the accumulation of unfolded proteins and elevate ER stress. The 78-kDa glucose-regulated protein (GRP78) is a major ER chaperone and suggested to act as primary sensor of unfolded protein load to activate the unfolded protein response (UPR), with protein kinase RNA-like endoplasmic reticulum kinase (PERK) mediating a major UPR arm by phosphorylation eIF2a to reduce protein translation as well as to activate transcriptional responses to regain homeostasis to promote apoptosis [46,47]. To establish that the ErbB2-overexpressing breast cancer cell lines SKBR3 and 21MT1 have an inducible ER stress pathway, we treated these cells with thapsigargin, a commonly used ER stress inducer [48]. Indeed, thapsigargin treatment induced an increase in the level of PERK phosphorylation as seen by an upward shift in the PERK band; the mobility was eliminated by treating the lysates with lambda phosphatase, validating its basis due to phosphorylation as a readout of PERK activation (Figure 4A and Figure S3A). Thapsigargin treatment also induced an increase in GRP78 levels (Figure 4A), further validating the induction of UPR in the ErbB2-overexpressing cell lines we use. To test the impact of CHIP levels in the context of ER stress, we treated SKBR3 and 21MT1 control and CHIP KD or overexpression derivative cell lines with thapsigargin. As shown in Figure 4B and Figure S3B and Figure 4C and Figure S3C, CHIP KD cells showed more prominent GRP78 induction and PERK phosphorylation together with increased phospho-eIF2α compared with control cells, suggesting that cells with lower CHIP levels are more prone to ER stress induction. Notably, CHIP overexpression cells showed a robust GRP78 induction, but substantially less PERK activation as well as less induction of phospho-eIF2α levels. While the molecular basis of the CHIP level-dependent differences in UPR will require future studies, the higher level of PERK activation in CHIP KD cells supports the idea that UPR induction by clinically used drugs could provide a potential approach to inhibit the growth of CHIP-low ErbB2-overexpressing tumor cells. To test this, we first performed soft agar colony formation assays to assess thapsigargin-induced tumor inhibition and the impact of CHIP levels. As shown in Figure 4D, both SKBR3 and 21MT1 cells showed thapsigargin dose-dependent growth inhibition that was sensitized by CHIP KD. The growth of CHIP-overexpressing SKBR3 cells, on the other hand, was inhibited to a lesser robust degree by thapsigargin, although this was not seen with 21MT1 cells. Overall, these results support the idea that reduced levels of CHIP, as are now known to occur in tumors such as ErbB2-overexpressing breast cancer [35,36,49], could render tumor cells more prone to ER stress and provide a basis for therapeutic targeting using clinically safe drugs with ER stress-inducing activity. 

### 3.5. Bortezomib Combined with Trastuzumab Synergistically Inhibits ErbB2-Overexpressing Breast Cancer Cell Proliferation 

**Figure 5:**
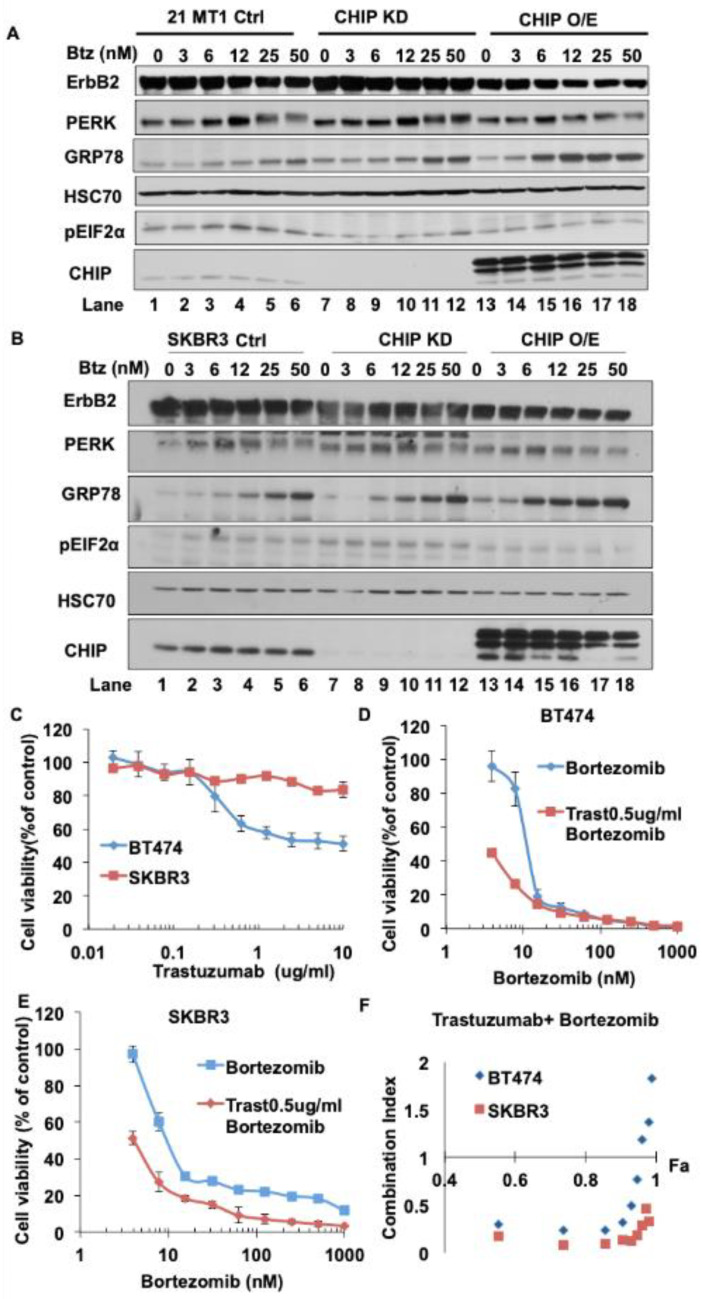
Bortezomib induces ER stress in ErbB2-overexpressing breast cancer cell lines and exhibits synergistic inhibition of cell proliferation when combined with Trastuzumab. (**A**,**B**) 21MT1 (**A**) and SKBR3 (**B**) cells were seeded in six-well plates and incubated with Bortezomib for 24 h. Cleared lysates (50 μg/lane) were subjected to Western blotting for ErbB2, GRP78, phospho-eIF2α, PERK, CHIP and Hsc70 (loading control). (**C**–**E**) BT474 and SKBR3 cells were seeded in 96-well plates and cultured for 5 days in the presence of either Trastuzumab or Bortezomib alone or combinations of the two at various concentrations. Viable cells were stained with the MTT dye and optical density read at 570 nm wavelength in a plate reader. Data represent mean ± SD, *n* = 3. (**F**) Combination index was calculated using the CalcuSyn software (http://www.biosoft.com/w/calcusyn.htm; accessed on 16 July 2021 per the method of T.C. Chou and Mike Hayball).

Depending on the duration and degree of ER stress, the UPR could provide either survival signals by activating adaptive and anti-apoptotic pathways, or death signals by inducing cell death programs. The former is thought to occur in tumor cells, which activate the UPR gradually and this contributes to their ability to survive and exhibit other oncogenic traits under hostile environments, such as tissue hypoxia and lack of nutrients [50,51]. Therefore, it has been proposed that acute elevation of ER stress or repression of the adaptive UPR mechanisms pharmacologically could elevate the ER stress to levels that produce cell growth inhibition or death, and hence beneficial therapeutic effects against cancer cells [52]. Given the above results with thapsigargin, we tested whether lower levels of CHIP expression in ErbB2-overexpresisng breast cancer cells could indeed sensitize them to acute elevation of ER stress with Bortezomib, a clinically used drug known to induce ER stress [49,53,54] and lead to an anti-tumor effect. As shown in Figure 5A,B (Figure S4A,B), similar to thapsigargin treatment, CHIP KD cells showed more PERK activation, higher phospho-eIF2α and more GRP78 induction compared with control cells upon Bortezomib treatment. CHIP overexpression cells showed more GRP78 induction but less PERK activation and lower phospho-eIF2α levels upon Bortezomib treatment. Collectively, these results demonstrate that Bortezomib could indeed elevate ER stress in ErbB2-overexpressing breast cancer cells and that this effect was more pronounced in CHIP-low cells, supporting the rationale to test the effect of this inhibitor against control vs. CHIP KD tumor cells.

The humanized monoclonal antibody Trastuzumab is used as a standard targeted therapy for ErbB2-overexpressing breast cancer patients. We therefore tested whether the elevated ER stress induction by Bortezomib could have a synergistic or additive effect with Trastuzumab against ErbB2-overexpressing, low CHIP-expressing, BT474 and SKBR3 breast cancer cells. As shown in Figure 5C–E, BT474 and SKBR3 cells showed a dose-dependent growth inhibition upon treatment with Bortezomib or Trastuzumab alone. Chou-Talaly analysis [55] of the Combination Index of the effects of combined Bortezomib and Trastuzumab treatment as an indicator of their interaction showed that Bortezomib and Trastuzumab synergize to inhibit BT474 and SKBR3 cell growth (combination indices between 0 and 1) (Figure 5F). These results further establish that CHIP is a key regulator of oncogenic traits and therapeutic sensitivity of tumor cells, and the results described here could form the basis of future efforts to target CHIP-low ErbB2-overexpressing breast cancers with a combination of ER stress inducers such as Bortezomib together with Trastuzumab to improve the response of patients with ErbB2-overexpressing breast cancer to ErbB2-targeted therapy.

## 4. Discussion

In this study, we establish a novel role of CHIP/STUB1 ubiquitin ligase in regulating ErbB2 by targeting it for degradation at an early post-biosynthesis step prior to the exit of mature ErbB2 from the Golgi. Our results suggest that CHIP-dependent alterations in the association of ErbB2 with molecular chaperones are an important mechanism to promote the Golgi to surface transport of newly synthesized ErbB2 in ErbB2-overexpressing breast cancer cells. These findings imply that loss of CHIP, which is seen in a majority of EbB2-overexpressing breast cancers [35], accentuates ErbB2-driven oncogenesis in part by ensuring that HSP90 remains associated with ErbB2 and allows this complex to exit the ER/Golgi for transport to the cell surface where it functions to promote oncogenesis.

Hsp90-Hsc70 chaperone monitors the folding state of the cytoplasmic domains of TM proteins [56,57]. CHIP, as a negative co-chaperone, promotes the pro-degradation state of the Hsp90-Hsc70 chaperone [45,58,59]. Studies of model TM proteins, such as CFTR, have demonstrated that misfolding of cytoplasmic domains triggers Hsp90-Hsc70 chaperone-dependent degradation apparently through ERAD [19,21]. Overexpressed ErbB2, which lacks any mutations, is persistently bound to Hsp90-Hsc70 chaperone through a unique hydrophobic patch on ErbB2 [24]. Inhibition of this association (by Hsp90 inhibitors) leads to rapid degradation of ErbB2 [29,30]. When the hydrophobic patch in ErbB2 was rendered EGFR-like, the mature form of this mutant ErbB2 was shown to be insensitive to Hsp90 inhibitors; under these conditions, Hsp90-Hsc70 still interacted with the cytoplasmic region, but through another undefined region, and Hsp90 inhibition only destabilized the newly synthesized form of ErbB2, indicating that Hsp90-Hsc70 chaperone was separately needed to stabilize the cytoplasmic domain of newly synthesized ErbB2. As shown in this study, CHIP plays an essential role as an enforcer of early post-biosynthetic degradation of ErbB2 in ErbB2-driven breast cancer lines. As CHIP-dependent degradation was enforced on immature ErbB2 and was accentuated by the retention of this form in the ER using brefeldin-A (Figure 2D,E), it appears to be consistent with the ER quality control roles assigned to CHIP in the context of mutant CFTR via ERAD [19]. VCP/p97 AAA ATPase is known to play an essential role in ERAD by coordinating the translocation of unfolded proteins with their ubiquitination or deubiquitination, through associated ubiquitin ligases and deubiquitinases [41]. Notably, however, inhibition of VCP/p97 using Eeyarestatin I (Figure 2F) led to an increase in the level of ubiquitination of ErbB2 and a reduction in its levels irrespective of CHIP expression status. These results suggest that VCP/p97 may help protect newly synthesized ErbB2, potentially due to deubiquitination, rather than subject it to ERAD. While additional studies will be needed to fully establish the potential role of VCP/p97 in ErbB2 quality control at the ER, it appears that a potential role of ERAD of ErbB2 is likely to be complex. Additionally, an example of the ER “quantity” control has been presented in which the ER-localized ubiquitin ligase Nrdp1 enforces the ERAD of ErbB3 to control its surface levels [15,16]. It is therefore plausible that CHIP-dependent control of the early post-biosynthetic degradation of ErbB2 could also function as a “quantity” control rather than a “quality” control since ErbB2 is transported to the cell surface in a chaperone-associated form and is functional in this form.

The classical ‘ERAD’ is thought to take place at the ER [60]. Since we observed the CHIP-mediated destabilization of immature ErbB2 in stably CHIP-overexpressing cells, yet intracellular ErbB2 was only seen in the Golgi in these cells (Figure 3B,D), the CHIP-dependent control of newly synthesized ErbB2 does not appear to simply involve retention in the ER followed by ERAD. It is therefore plausible that CHIP may function at both organelles during the quality/quantity control of newly synthesized TM proteins such as ErbB2. Since our results with pulse-chase labeling clearly showed that CHIP regulates the conversion of newly synthesized immature to mature form of ErbB2 and that inhibition of ER to Golgi transport of ErbB2 exposed the immature ErbB2 to CHIP-dependent degradation (Figure 2B–E), we suggest that CHIP function towards ErbB2, and potentially other TM proteins, is more complex than simple ERAD. We speculate that CHIP association with molecular chaperones bound to TM proteins such as ErbB2 promotes ERAD and in addition can expose the targeted proteins for degradation even in non-ER compartments such as Golgi, but that such processes may be slower compared to ERAD. This could account for our observation of a pool of CHIP and ErbB2 in the Golgi but not in the ER in stably CHIP-overexpressing ErbB2-overexpressing breast cancer cells. This idea is consistent with recent identification of endosome/Golgi-associated degradation pathway in yeast [42] and with reports in which mutant CFTR located at the cell surface was shown to undergo an ERAD-like degradation dependent in part on CHIP [61]. It is also possible that CHIP-dependent ubiquitination of either the molecular chaperones [62] or the TM proteins such as ErbB2 (Figure 2A) [29,30] promotes retrograde transport of the latter from the Golgi to the ER where the marked proteins are then eliminated by ERAD. Notably, Carboxypeptidase Y (CPY) protein has been shown to be retro-transported from the Golgi to the ER for ERAD [63]. We speculate that partially unfolded ErbB2, apparently in association with Hsp90 can progress to the Golgi apparatus. However, the association of CHIP with molecular chaperones is likely to prevent the further transport of ErbB2 and may promote retro-transport to the ER for degradation. Notably, overexpressed ErbB2 is unique among its family members to require Hsp90 for the stability of its mature form, including that present on the cell surface; in contrast, other RTKs, such as EGFR, require Hsp90 only in their newly synthesized forms [57]. In a previous study from our laboratory [29], elevated interaction of ErbB2 and Hsp70 was found upon U-box mutant CHIP H260Q expression, but ErbB2/Hsp90 complex did not disassociate when cells were treated with an Hsp90 inhibitor 17AAG, a result that is in contrast to results with parental cells or cells expressing wildtype CHIP. We reason that CHIP, as a negative co-chaperone for Hsp90/Hsc70 [32], promotes the release of Hsp90/Hsc70 from ErbB2, thereby exposing the inherent hydrophobic sequences previously identified in ErbB2 to be required for Hsp90 chaperone binding [24]. It is likely that this switch leads to the newly synthesized ErbB2 being sensed as an unfolded protein, promoting its retention in the Golgi/ER and eventual targeting for degradation by ERAD or a modification of this pathway. Thus, tumor-associated loss of CHIP can be viewed as an adaptive mechanism to relieve ErbB2 associated with Hsp90 of a bottleneck on its transit to the cell surface.

The demonstration of CHIP as an enforcer of ErbB2 degradation during the post-biosynthesis phase, potentially involving ERAD, should allow future studies to test therapeutic options that target components of ERAD and the linked unfolded protein stress and UPR pathways. Recent studies have shown that targeting the UPR pathway could sensitize ErbB2-directed therapies such as trastuzumab [64,65,66]. It was reported that Bortezomib, which is FDA-approved for the treatment of multiple myeloma [67], has a very potent inhibitory effect on breast cancer cell lines, and clinical trials to test Bortezomib in breast cancer patients are ongoing (ClinicalTrials.gov Identifier: NCT04265872). We confirmed that Bortezomib, similar to experimental tool agent thapsigargin, induced the ER stress in ErbB2-overexpressing breast cancer cell lines and that CHIP KD cells exhibited higher sensitivity to thapsigargin or Bortezomib (Figure 4D). Our in vitro proliferation data (Figure 5D,E) demonstrated synergistic inhibition of proliferation of ErbB2-overexpressing breast cancer cell lines with Bortezomib and Trastuzumab. Together, these results suggested that a combination of Bortezomib and Trastuzumab could produce a therapeutic improvement specifically in ErbB2-overexpressing patients that show reduced CHIP expression. It will be of considerable value to assess whether low CHIP expression may serve as a correlative marker for Bortezomib plus Trastuzumab responsiveness in ErbB2-overexpressing breast cancer.

## 5. Conclusions

ErbB2 is amplified or overexpressed in up to 25% of human breast cancers. Display of overexpressed ErbB2 at the cell surface following its biosynthesis in the endoplasmic reticulum is required for oncogenesis, but mechanisms that regulate how this process is optimized in tumor cells are poorly understood. Since ErbB2 requires continuous association with HSP90 molecular chaperone for its stability and function as an oncogenic driver, the authors hypothesized that the HSP90/HSC70-interacting negative co-chaperone and E3 ubiquitin ligase CHIP/STUB1, whose expression is lost in most ErbB2-overexpressing breast cancers, provides one such mechanism. The present work shows that CHIP targets the newly synthesized, HSP90/HSC70-associated ErbB2 for ubiquitin/proteasome-dependent degradation in the endoplasmic reticulum and Golgi, thus identifying a novel mechanism that negatively regulates the cell surface display of overexpressed ErbB2 levels in breast cancer cells, explaining in part the frequency of CHIP expression in ErbB2-overexpressing breast cancers. The current studies also reveal that ErbB2-overexpressing breast cancer cells with low CHIP expression exhibit higher induction of endoplasmic reticulum stress. Accordingly, the endoplasmic reticulum stress-inducing anticancer drug Bortezomib showed synergistic inhibition of ErbB2-overexpressing breast cancer cell proliferation when combined with ErbB2-targeted humanized antibody Trastuzumab. The new insights into mechanisms that control the surface expression of overexpressed ErbB2 identified here support the potential of exploring combined treatment with Trastuzumab and ER stress-inducing agents in ErbB2-overexpressing breast cancers with loss of CHIP expression.

## Data Availability

Not applicable.

## Supplementary Materials

The following are available online at https://www.mdpi.com/article/10.3390/cancers13163936/s1, Figure S1: CHIP regulates cell surface ErbB2 levels, Figure S2: CHIP elevates the basal ubiquitination of ErbB2 during its maturation, Figure S3: Reduced expression of CHIP sensitizes ErbB2-overexpressing breast cancer cell lines to ER stress, Figure S4: Bortezomib induces ER stress in ErbB2-overexpressing breast cancer cell lines.
