# Supplementary material for: CHIP/STUB1 Ubiquitin Ligase Functions as a Negative Regulator of ErbB2 by Promoting Its Early Post-Biosynthesis Degradation
Source: Cancers (Basel). 2021 Aug 4;13(16):3936. doi: 10.3390/cancers13163936 (PMC8391510; doi:10.3390/cancers13163936)
Supplement: Supplementary file 1 [file cancers-13-03936-s001.zip › cancers-1322707-supplementary.pdf]

Supplementary Files

CHIP/STUB1 Ubiquitin Ligase Functions as a Negative Regulator of ErbB2 by Promoting Its Early Post-Biosynthesis Degradation

Haitao Luan, Tameka A. Bailey, Robert J. Clubb, Bhopal C. Mohapatra, Aaqib M. Bhat, Sukanya Chakraborty, Namista Islam, Insha Mushtaq, Matthew D. Storck, Srikumar M. Raja, Vimla Band and Hamid Band

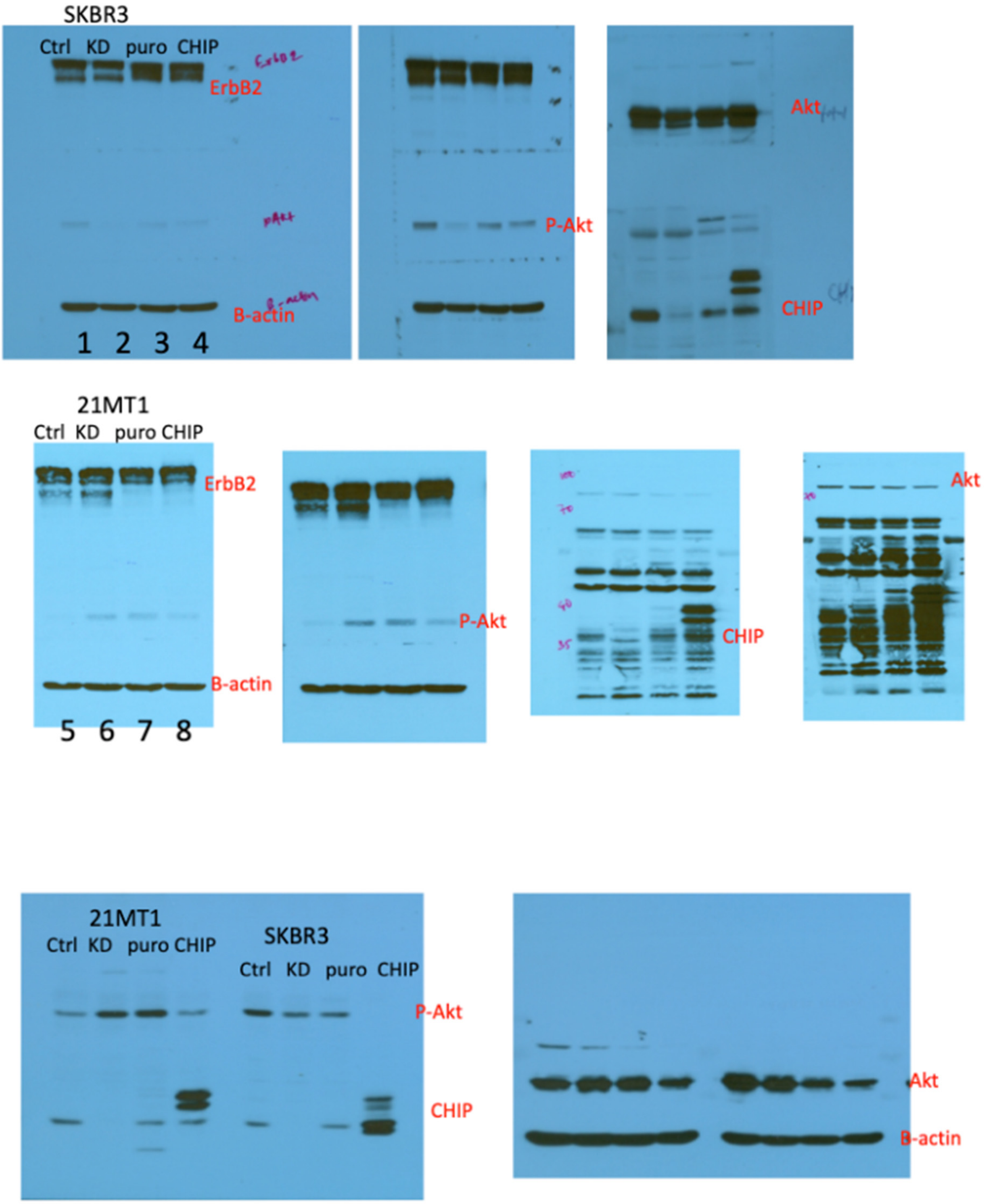

Figure S1. CHIP regulates cell surface ErbB2 levels. Original blots of western blot analysis of SKBR3 and 21MT1 cell lyScheme 2. phospho-Akt and total Akt levels, and β-actin as a loading control.

(A)

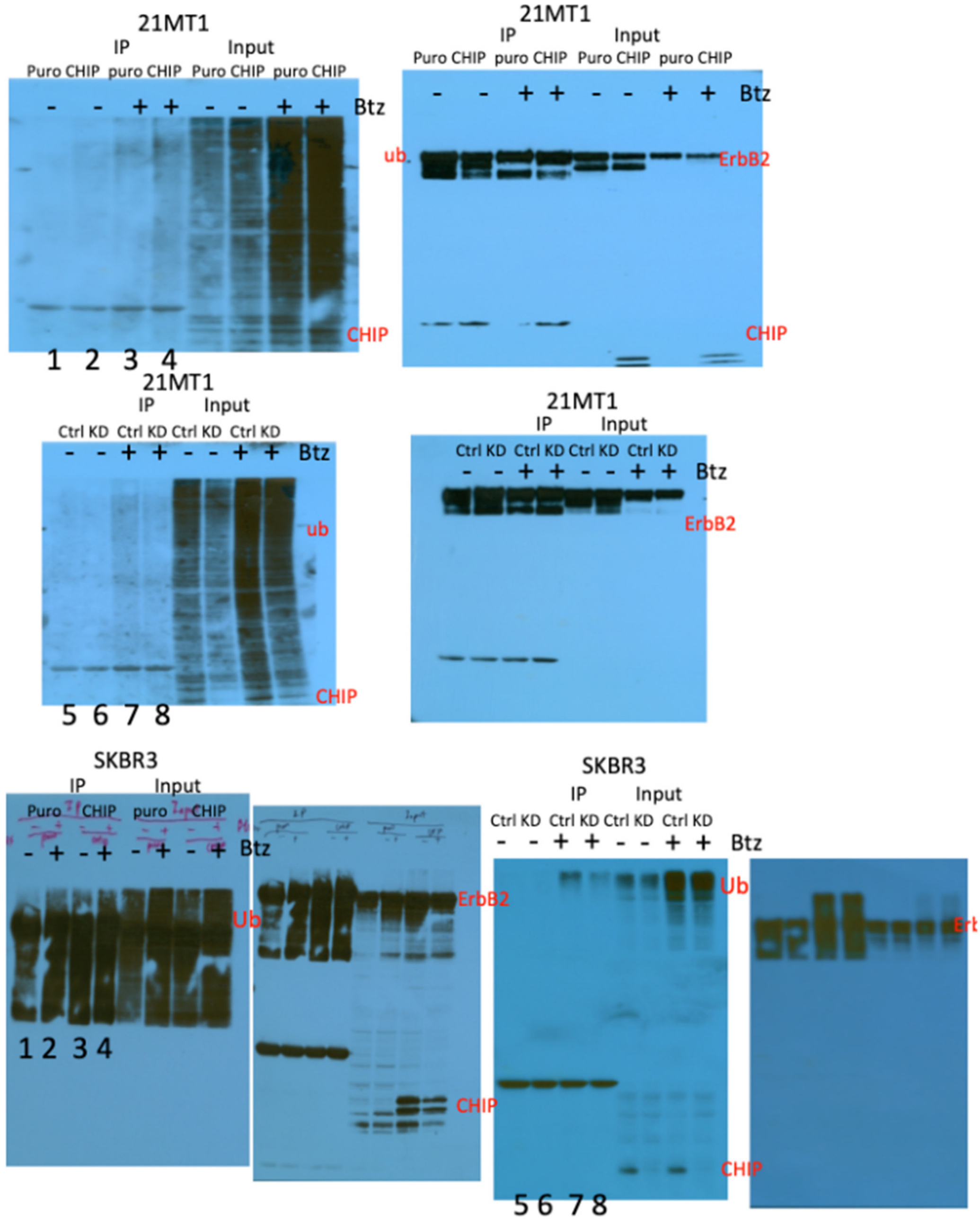
21MT1
IP
Input
Puro CHIP puro CHIP Puro CHIP puro CHIP
- - + + - - + + Btz
CHIP
1 2 3 4
ub
ErbB2
21MT1
IP
Input
Ctrl KD Ctrl KD Ctrl KD Ctrl KD
- - + + - - + + Btz
ub
CHIP
5 6 7 8
ErbB2
SKBR3
IP
Input
Puro CHIP puro CHIP
- + - + - + - + Btz
Ub
1 2 3 4
ErbB2
CHIP
SKBR3
IP
Input
Ctrl KD Ctrl KD Ctrl KD Ctrl KD
- - + + - - + + Btz
Ub
CHIP
5 6 7 8

(B)

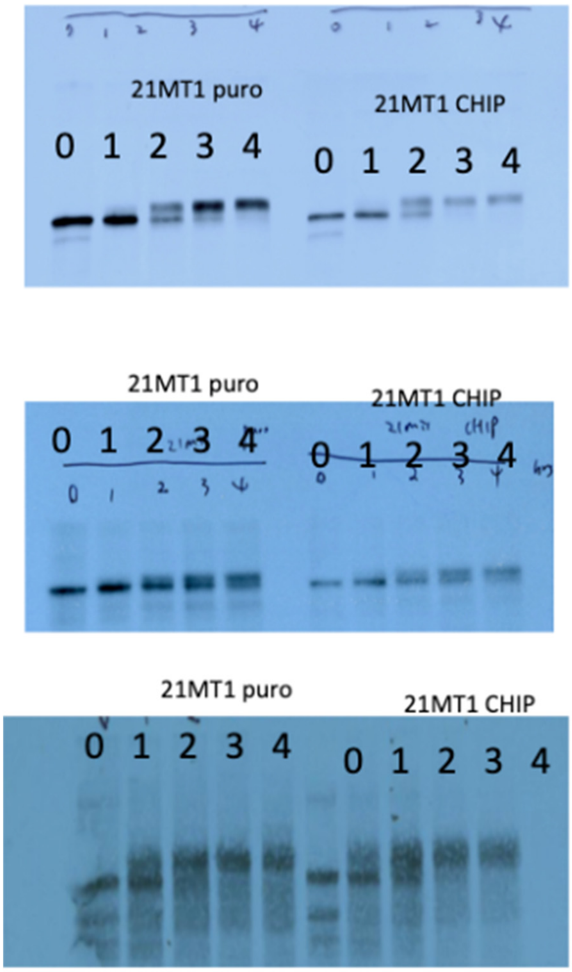
21MT1 puro
21MT1 CHIP
0 1 2 3 4
0 1 2 3 4
21MT1 puro
21MT1 CHIP
0 1 2 3 4
0 1 2 3 4
21MT1 puro
21MT1 CHIP
0 1 2 3 4
0 1 2 3 4

(**C**)

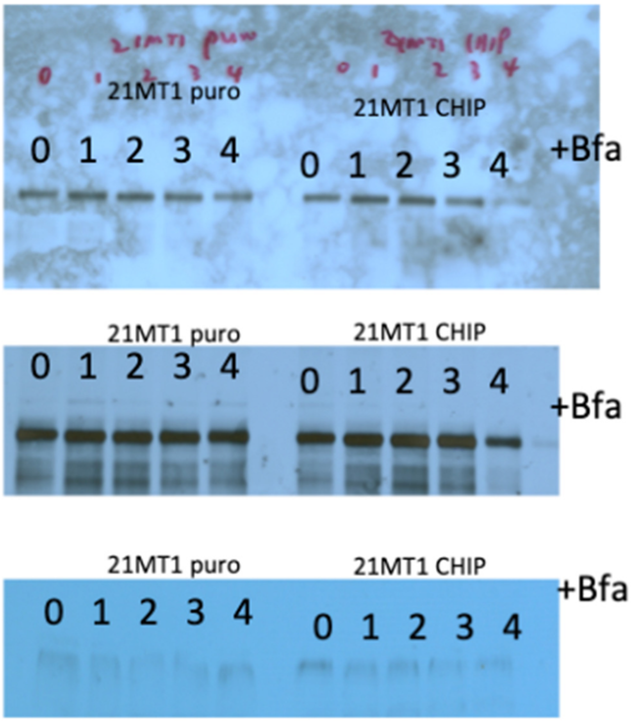
21MT1 puro
21MT1 CHIP
0 1 2 3 4
0 1 2 3 4
+Bfa
21MT1 puro
21MT1 CHIP
0 1 2 3 4
0 1 2 3 4
+Bfa
21MT1 puro
21MT1 CHIP
+Bfa
0 1 2 3 4
0 1 2 3 4

(**D**)

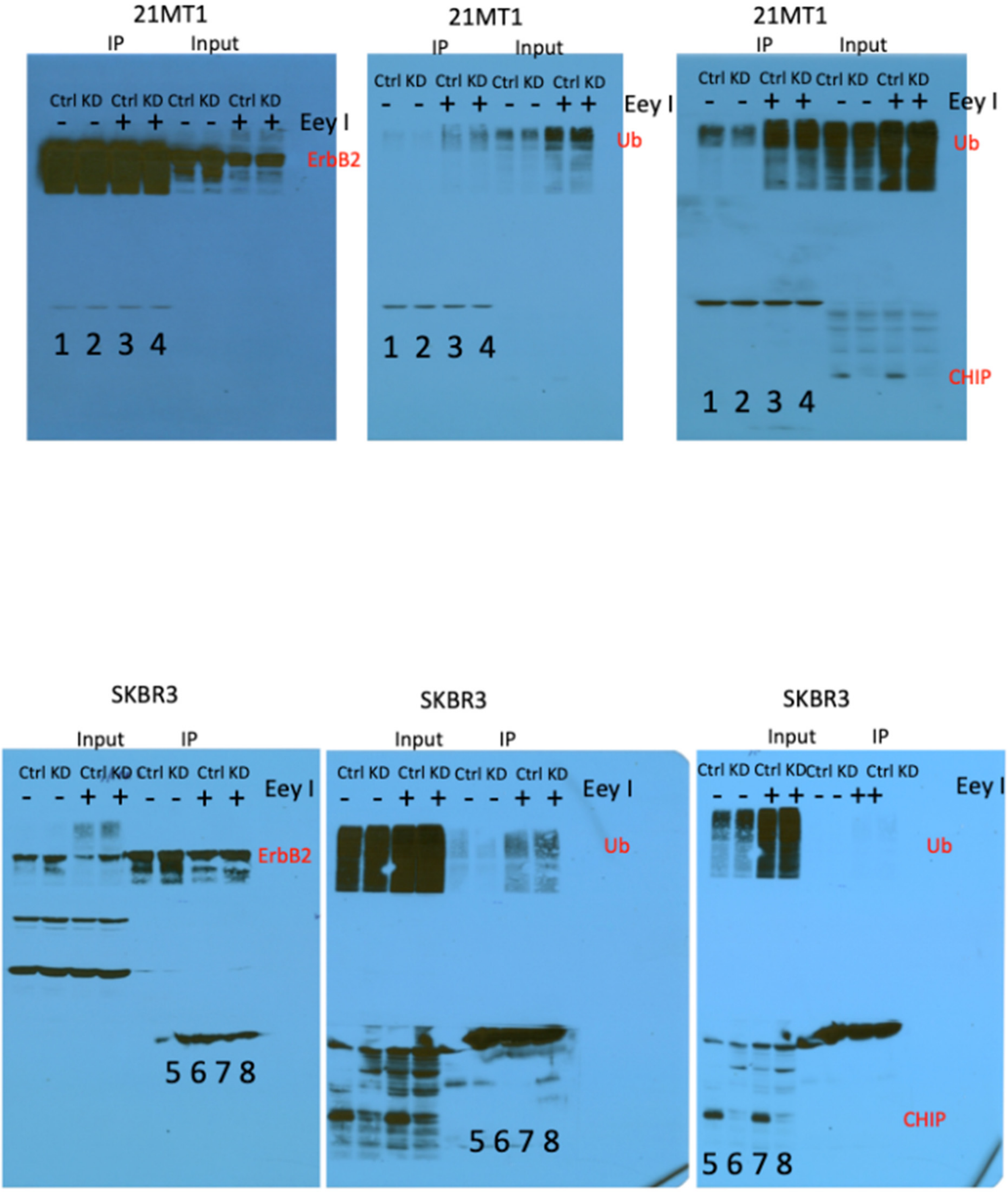

Figure S2. CHIP elevates the basal ubiquitination of ErbB2 during its maturation. (**A**) Original blots of immunoprecipitation of ErbB2 in 21MT1 and SKBR3 cells treated with Bortezomib (1 μM) for 4h. (**B**) Original blots of [^{35}S]-methionine/cysteine chase of ErbB2 in 21MT1. (**C**) Original blots of [^{35}S]-methionine/cysteine chase of ErbB2 in 21MT1 with Brefeldin A (1 μg/mL). (**D**) Original blots of immunoprecipitation of ErbB2 in 21MT1 and SKBR3 cells treated with Eeyarestatin I (1 μM) for 4 h.

.

(**A**)

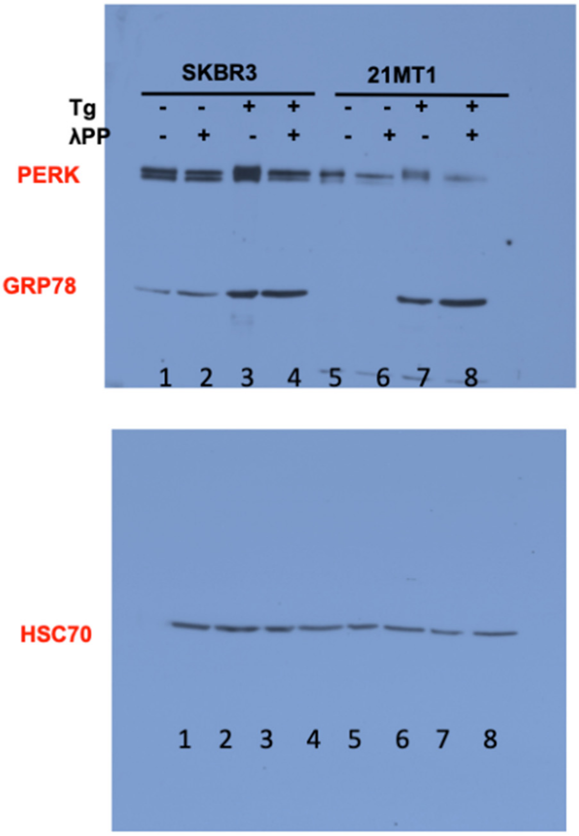

(**B**)

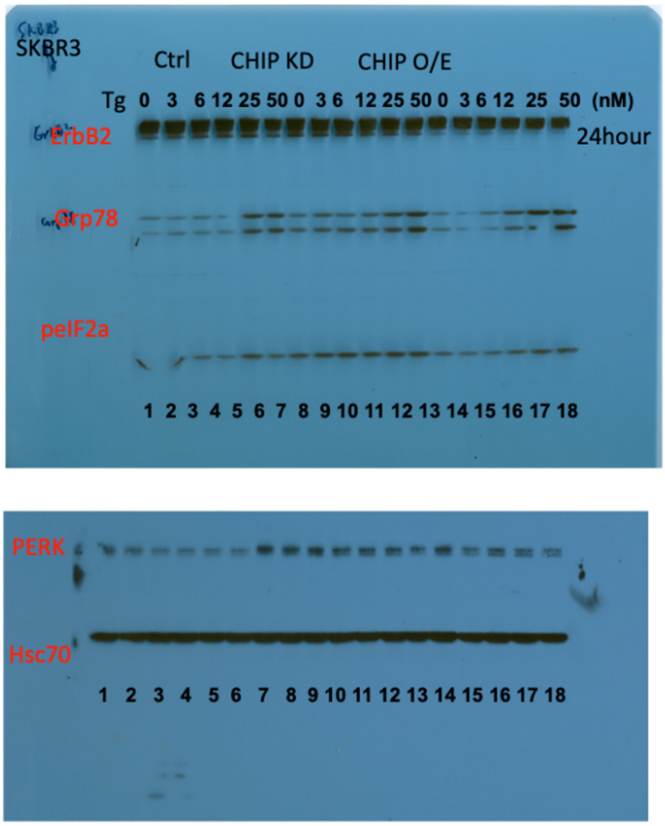
SKBR3
Ctrl
CHIP KD
CHIP O/E
Tg 0 3 6 12 25 50 0 3 6 12 25 50 0 3 6 12 25 50 (nM)
ErbB2
24hour
Grp78
peIF2a
1 2 3 4 5 6 7 8 9 10 11 12 13 14 15 16 17 18
PERK
Hsc70
1 2 3 4 5 6 7 8 9 10 11 12 13 14 15 16 17 18

(C)

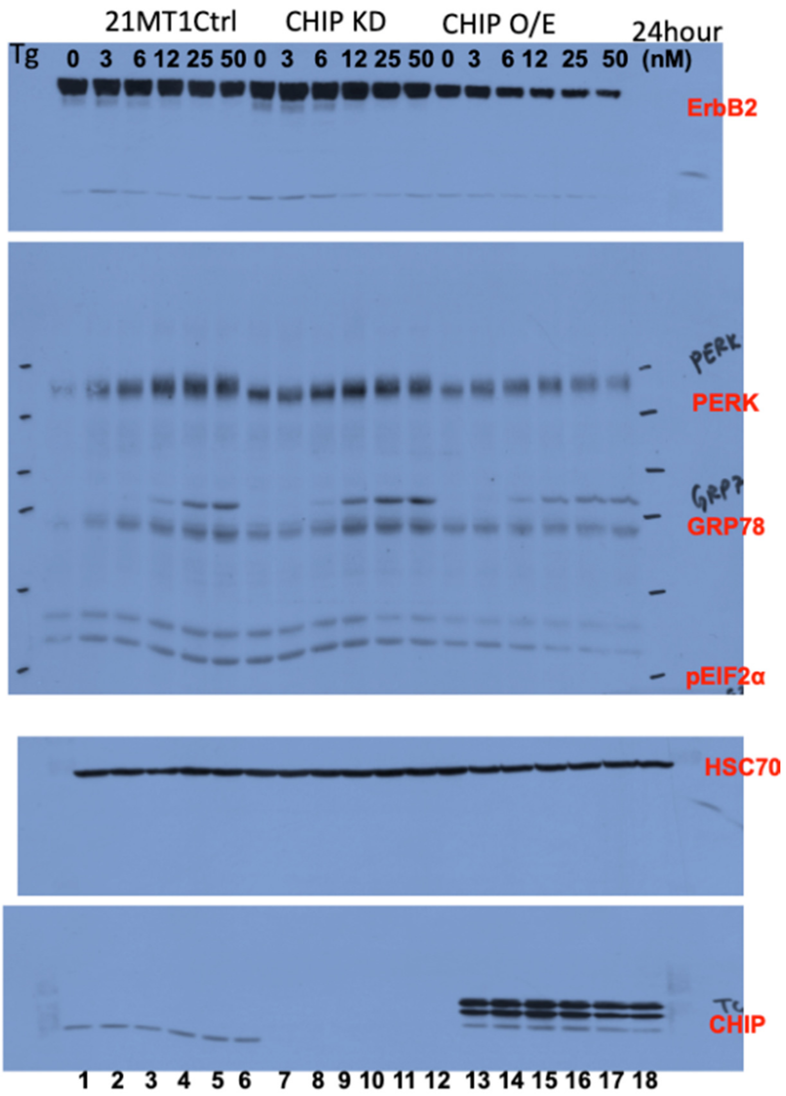

Figure S3. Reduced expression of CHIP sensitizes ErbB2-overexpressing breast cancer cell lines to ER stress. (**A**) Original blots of western blot analysis of SKBR3 and 21MT1 cell treated with thapsigargin (50 nM) for GRP78 and PERK. (**B**) Original blots of western blot analysis of SKBR3 cell with thapsigargin treatment for ER stress markers. (**C**) Original blots of western blot analysis of 21MT1 cell treated with thapsigargin for ER stress markers.

(A)

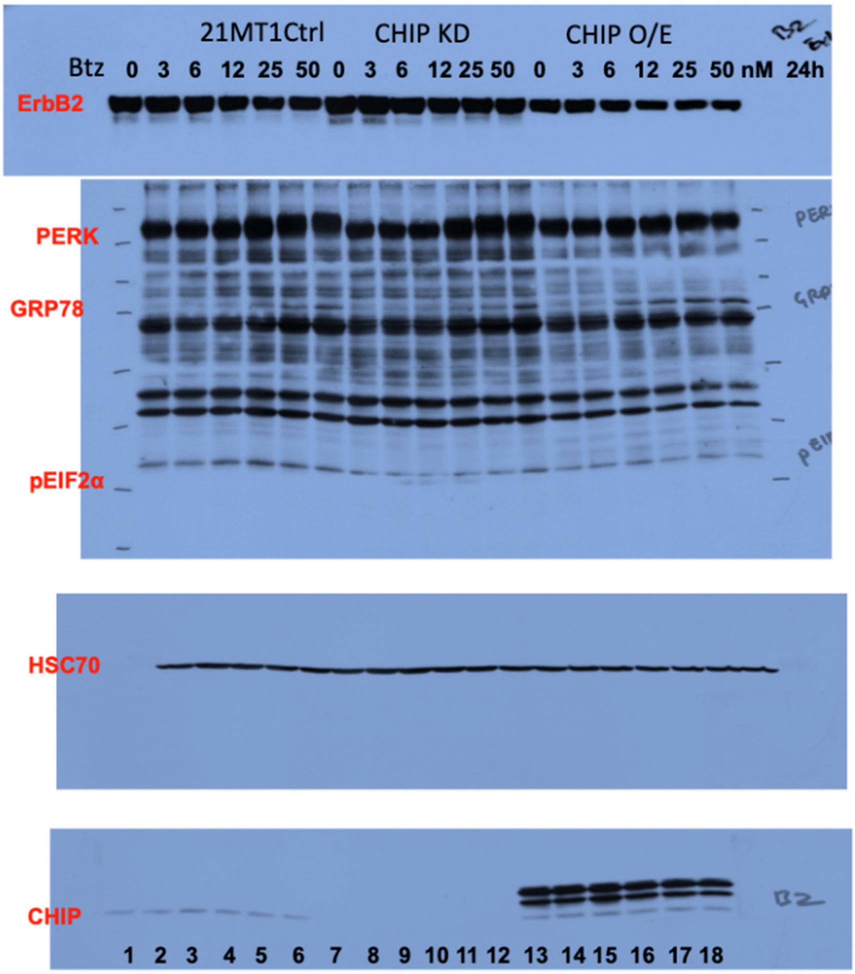
21MT1Ctrl
CHIP KD
CHIP O/E
Btz 0 3 6 12 25 50 0 3 6 12 25 50 0 3 6 12 25 50 nM 24h
ErbB2
PERK
GRP78
pEIF2α
HSC70
CHIP
1 2 3 4 5 6 7 8 9 10 11 12 13 14 15 16 17 18

(B)

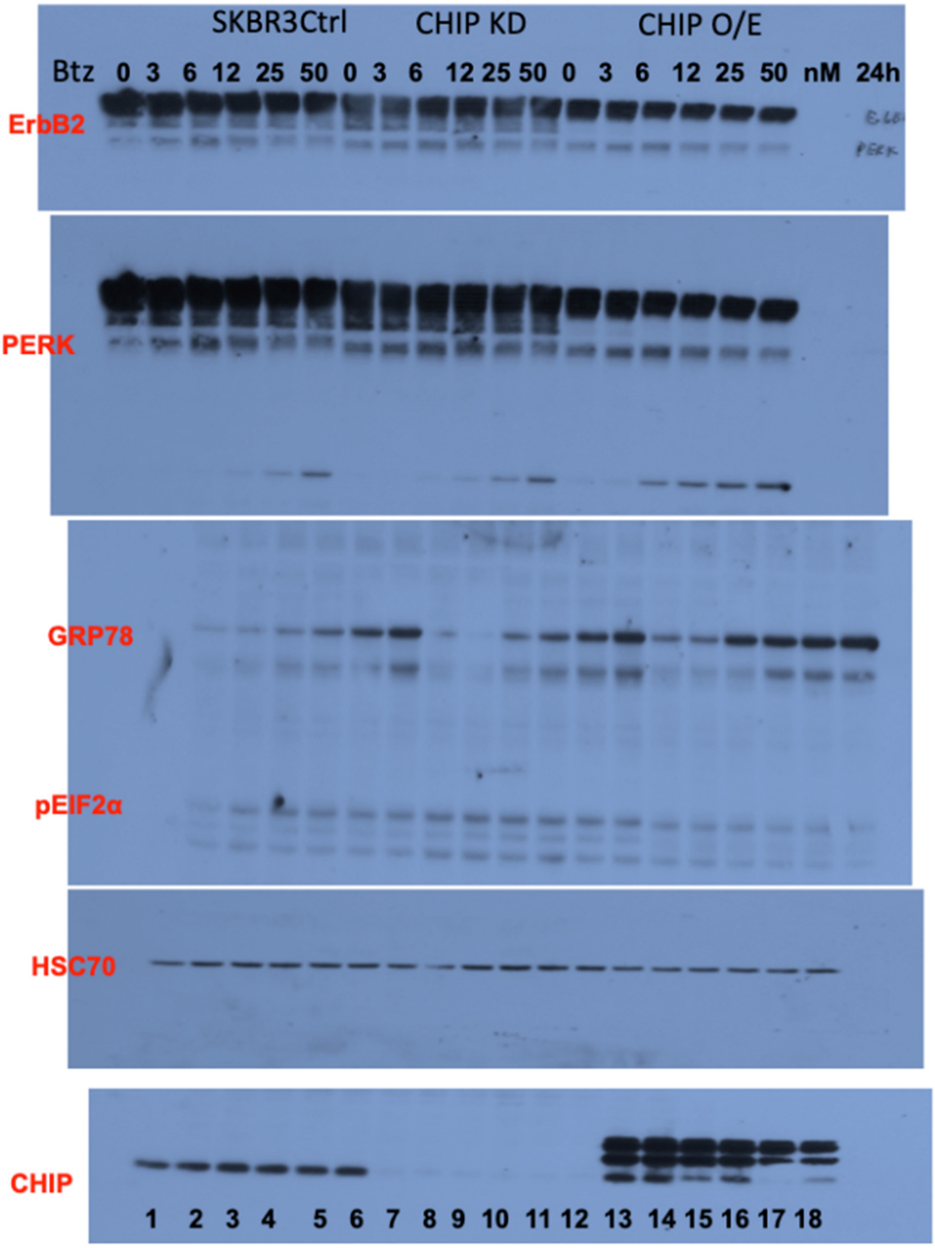

Figure S4. Bortezomib induces ER stress in ErbB2-overexpressing breast cancer cell lines. (**A**) Original blots of western blot analysis of 21MT1 cell treated with Bortezomib treatment for ER stress markers. (**B**) Original blots of western blot analysis of 21MT1 cell treated with Bortezomib treatment for ER stress markers.
